# Enhancing critical thinking skills and media literacy in initial vocational education and training *via* self-nudging: The contribution of NERDVET project

**DOI:** 10.3389/fpsyg.2022.935673

**Published:** 2022-08-05

**Authors:** Riccardo Sartori, Francesco Tommasi, Andrea Ceschi, Mattia Falser, Silvia Genero, Silvia Belotto

**Affiliations:** ^1^Department of Human Science, University of Verona, Verona, Italy; ^2^ENAIP Veneto Foundation, Padova, Italy

**Keywords:** critical thinking skills, media literacy, self-nudging, vocational education and training (VET), NERDVET

## Abstract

Vocational Education and Training (VET) programs are fuelled by technical and practical educational modules. The teaching staff adopts both traditional and innovative pedagogical frameworks to increase the generalization and maintenance of practical skills. At the same time, VET teachers and trainers have a few occasions to promote and include disciplines and educational programs for enhancing students' soft skills, e.g., critical thinking skills (CT) and media literacy (ML). Following the European VET framework and literature of the field, CT and ML represent a social challenge that requires even more efforts by academics, practitioners, and policymakers. Thisstudy situates into this context with the aim of introducing a novel educational approach for supporting the teaching staff in the promotion of students' CT and ML. This educational approach has been realized by the team of researchers and trainers of the NERDVET project, an Erasmus+ KA3 project devoted to the promotion of new tools and policies for enhancing CT and ML in VET. To pursue this aim, the team has employed the self-nudging model which regards the individuals' set of cognitive and behavioral strategies that individuals can develop to target a specific objective. By framing pedagogical strategies into this perspective, the team realized an initial approach for educational activities and teaching strategies to promote students' CT and ML.

## Introduction

Vocational education and training (VET) programs aim at equipping students and learners with a supply of technical and practical skills aligned with the labor market's needs. This is notable not only in VET pedagogical frameworks, and in the choice of educational modules of VET providers, but also more institutionally in normative definitions and operationalization of VET centers. This is due to the nature of the purpose of VET to equip students with skills as the glue in between the new workforce and the productivity of specific working sectors. The transformations concerning the labor market underline that the labor market benefits more from the VET sector than other educational pathways. However, this entails that the promotion of technical and practical skills can be insufficient with respect to the promotion of additional skills, e.g., critical thinking skills and media literacy. Focusing more on technical skills at the expense of metacognitive skills may compromise individuals' citizenship behavior (Pfaff-Rüdiger and Riesmeyer, 2016; Tommasi et al., 2021a; Perini et al., 2022). The lack of educational models for the promotion of metacognitive competences in VET-led scholarly authors, practitioners, and policymakers to move toward the creation of pathways aimed at the development of these specific components. For example, the European Union (EU) has made skills like critical thinking and media literacy key objectives for the education and training sectors. Following this trend, the EU countries and the European Commission (EC) have used and financed multiple initiatives (e.g., Erasmus+, the Connecting Europe Facility, (European Commission, 2020b)).

It is in this context that the *Think smart! Enhancing critical thinking skills and media literacy in VET* (NERDVET, n.d.) project, an Erasmus+ KA3 project co-funded by the European Commission^1^, takes place with the proposition of developing a novel educational program to support VET teaching staff in increasing CT and ML skills of their students. The NERDVET educational program is based on different techniques, among which a novel concept of self-nudging has been developed: according to this new self-nudging concept, teachers and trainers can foster students' capacity to create a set of specific personal strategies to reach a target or to tailor their behavior for a proactive purpose, e.g., behaving critically in a digital environment. Through self-nudging, it is possible to develop the proactive commitment of individuals in the processing of information, also aiming at supporting the creation of specific individuals' strategies to critically evaluate information and adopt a specific behavior.

The aim of this study is to present the NERDVET proposal to use the self-nudging model for enhancing students' critical thinking skills and media literacy. At the base (i.e., ontologically, Creswell, 2014), critical thinking and media literacy represent two linked metacognitive competences. On the one hand, critical thinking is a metacognitive competence concerning the knowledge and skills of reflection, analysis, and questioning of information, which results in proactive and citizenship behavior. On the other hand, media literacy as a metacognitive competence includes the knowledge and skills to think critically about media information through understanding media representations, structures, and implications (Tommasi et al., 2021a). Studying critical thinking and media literacy *via* a psychological (behavioral-cognitive) approach finds a connection with the notion of self-nudging, that is the individuals' own set of metacognitive strategies to pursue personal targets (Hertwig and Grüne-Yanoff, 2017; Torma et al., 2018). With this framework, we propose indications of possible ways through which teachers and trainers can enhance critical thinking and media literacy among VET students. Our indications serve to create the basis for realizing learning strategies to be implemented within the classroom. Ultimately, this proposal covers both the theoretical background and educational suggestions on implementing exercises whose purpose is the development of these metacognitive competences.

In the following sections, we will first report the current trends for the enhancement of CT and ML in the context of VET. Given the area of intervention of the NERDVET project, we will focus on the European trends for the enhancement of CT and ML in the VET context. Then, we will introduce how CT and ML are considered at the academic level. Here, we will report the definitions of CT and ML as well as an overview of the practices for the enhancement of CT and ML in VET. Second, considering these pieces of knowledge as a reference framework, we will report the self-nudging approach for the implementation of training techniques in the context of VET. We will refer to the ontological similarities between the notions of critical thinking, media literacy, and self-nudging theory to propose a novel approach serving learning strategies within the classroom. Lastly, we will end the discussion of the NERDVET approach for the enhancement of CT and ML by presenting the direct users and beneficiaries of this novel approach for training VET students.

## Approaches to critical thinking and media literacy

### European trends for the enhancement in the VET context

The integration of CT and ML in VET curricula is still very scant at the European level, although some preliminary initiatives have been carried out successfully in the last few years. The European institutions have introduced several policies and financial initiatives to support the goal of enhancing CT and ML in the context of vocational training, especially following the COVID-19 outbreak. This is the case of the EC Recommendation on Key Competences for Lifelong Learning, which has outlined a set of eight competencies that all individuals need for personal fulfillment and development, active citizenship, social inclusion, and employment. Similarly, the New Skills Agenda for Europe highlights 10 actions to make relevant training, skills, and support available to EU citizens. The European Trend 2020 strategic framework promotes peer learning, including through the collection and dissemination of good practices in the field of CT and ML, while paying special attention to effectively reaching out to disadvantaged learners and those at risk of marginalization. Also, the Commission's Digital Education Action Plan contains 11 actions to make better use of digital technology for teaching, learning, and developing digital competencies, based on the precondition that digital competence includes the confident, creative, and critical use of information and communications technology, which is also considered a crucial component of media literacy. To promote ML and CT, EU funds and programs, such as Erasmus+, the Connecting Europe Facility, the European Structural and Investment Funds, Horizon 2020, Creative Europe and Europe for Citizens, have been utilized by EU countries and the EC. Overall, experts in the field (e.g., policymakers, practitioners, and researchers) agree on the fact that “critical thinking is a widely accepted educational goal […] and its adoption as an educational goal has been recommended based on respect for students' autonomy and preparing students for success in life and democratic citizenship” (Hitchcock, 2018).

In contrast to this background, in the VET sector, there is little to no integration of critical thinking skills and media literacy within VET curricula or competence standards. In contrast to other countries (Decreto 220, 1998; Decreto 254, 2009; Australian Government Department of Education Training, 2016), VET curricula at the European level rarely contemplate systematic or integrated teaching of critical thinking either as specific content or as a transversal one (European Commission, 2020a). The organization of teaching sessions devoted to the development of such competences for students is thus left to VET schools, which–however–do not often have the means and opportunity to do so. Although some transversal skills related to critical thinking are embedded in different subjects and skills, they are neither sufficiently highlighted nor presented in a structured form. The largest part of learning projects remains grounded in implementations meant as a singular intervention and, even when it is not so, it tends to focus exclusively on specific aspects of critical thinking and media literacy. Quite often, these aspects are not treated in an integrated way, but their focus depends on the specific purpose of the project or lesson being carried out (Bergstrom et al., 2018).

### Overview of reference definitions and current practices

Notwithstanding the institutional context, academics in the field of VET have produced several contributions to CT and ML in recent years. The term *critical thinking* regards the human metacognitive ability to think clearly and rationally about something. Through critical thinking, individuals can (a) understand logical connections between ideas, (b) identify and evaluate arguments, (c) detect inconsistencies and common mistakes in reasoning and (d) achieve other fundamental aspects (e.g., daily decision-making process) (Kenyon, 2014; Bergstrom et al., 2018; Tommasi et al., 2021a,b). Critical thinking is also crucial to moving through the wide world of news that we read every day and avoiding judgment errors (Ceschi et al., 2019; Ceschi and Fioretti, 2021; Tommasi et al., 2021a,b,c). It also helps us to judge and understand a lot of aspects of what we read in the media. In this context, critical thinking is viewed at the same level of optimal decision-making competence which relates to the ability to avoid cognitive errors and the use of heuristics (Kenyon, 2014). Moreover, it is also an antecedent of positive social skills to critical thinking with issues such as body image, racial stereotypes, and gender (Bergstrom et al., 2018).

As for other terms such as *information* and *digital abilities* (Bolaños Cerda et al., 2020; Bolaños and Pilerot, 2021), media literacy is also characterized by terminological ambiguity as it has been discussed in a plurality of different forms with multiple arguments on how to improve it (van Laar et al., 2017; Bolaños et al., 2022). This partially reflects the lack of consensus on how to enhance media literacy among VET students, namely how to define media literacy in such context and to consider it in line with critical thinking. Arguments have been proposed that media literacy is linked to the notion of critical thinking and regards the ability to identify different types of media and understand the messages they are sending. Media literacy represents a core characteristic of citizenship behavior as well as an indispensable aspect for dealing with the huge amount of information presented in different shapes. Despite the agreement on its importance, authors reported different definitions and attributes of what media literacy means, such as the ability to critically access, analyze, evaluate, and create media messages (Banerjee et al., 2015; Schilder and Redmond, 2019). Other authors considered media literacy as the cognitive awareness of the importance of media messages and their impact on the public. Such awareness is meant to foster individuals' responsibility to critically evaluate media messages (Geers et al., 2020). Moreover, other authors considered media literacy as the ability to reach and understand the information within the media context, although the authors did not provide a clear idea about how such a process is sustained (Cohen and Mihailidis, 2013). In particular, they support the idea that individuals can make meaning of the contents and enhance their ability to make decisions.

However, there is still a certain degree of uncertainty concerning the agreement on what could be done to support teaching staff in the promotion of CT and ML in VET students. With respect to these, there are different ways of approaching CT and ML in VET. Some authors refer to the model of social interactions as a learning process to promote these competences (Bandura, 1986). This theory has been used to support the idea that CT and ML, as learning processes, may be the result of the vision and interaction between students and teachers (Banerjee et al., 2015). Other authors propose a broad social view of the importance of CT and ML, using the human capital theory model to sustain the need to actualize educational models on CT and ML in the context of VET (Edokpolor and Abusomwan, 2019). Similarly, others refer to Watson and Glaser (2002) model to argue that the promotion of critical thinking and media literacy is a consequence of a greater sense of belonging among students and, if promoted, also better active citizenship. Finally, through a cognitive approach, other authors use cognitive psychology models of the so-called debiasing as an intervention tool for the promotion of critical thinking and media literacy in the context of Initial VET (iVET) and citizenship behavior through teaching error recognition and cognitive distortions (Kenyon, 2014).

Such uncertainty is due to the multiplicity and complexity of factors interviewing on CT and ML, which makes it even more difficult to realize effective educational models for their promotion. Reviewing the literature in the field, Tommasi et al. (2021a) argued that there is a wide range of relevant factors for students, teachers, group-class, and communities that scholarly authors have been considering to propose training interventions. These include teaching techniques that, through stimulating reflectiveness, can help promote critical thinking and media literacy, also beyond the iVET context. Although there is not vast literature in this regard, this literature review suggests the existence of possible strategies which allow for improving individuals' disposal, personal resources, and reducing biases and cognitive prejudices (Noorani et al., 2019). In this regard, the teacher's role becomes crucial because they are called to set up the right conditions in the learning contexts to enhance those metacognitive skills. It is important to give appropriate input and tools that can support these processes to offer students the chance to keep implementing those metacognitive competences through personal and self-developed stimuli. Teachers may use significant samples, combined with the possible specific contextualization, to foster the comprehension of the consequent benefits of applying critical thinking and media literacy. In addition, this contribution aims to suggest learning practices and cues for personalizing the interventions toward the enhancement of critical thinking and media literacy in the context of iVET. This entails the importance of focusing on the individual and the comprehension of where to apply those skills in their specific situations.

## The NERDVET approach

Considering the very definitions and practices (Tommasi et al., 2021a), and the very institutional approaches as well, these can be interpreted through a behavioral-cognitive psychology approach and in particular *via* nudging and self-nudging models. At the base (i.e., ontologically, Creswell, 2014), critical thinking skills and media literacy regard how individuals understand information and concepts as well as promote specific cognitive and behavioral strategies. This echoes both the theoretical and empirical knowledge of the bunch of psychology and behavioral sciences devoted to the study of the human decision-making process (Cohen and Jaffray, 1980; Bell et al., 1988). In this, scholars have proposed an approach aiming to make ideal normative decision-makers, considering their cognitive limitations and trying to help them through the implementation of particularly difficult tasks and operations as a reframing action (Baron, 2000). Accordingly, individuals would be equipped with a set of logical abilities linked thanks to their reflective abilities in problem-solving activities, and their comprehension of the causes and effects of possible flawed choices. Trainers are asked to support the decision-making process of individuals with methods that help to reduce or eradicate errors. For example, the observation of commonly used reactions that are inconsistent with certain information for problem-solving can be considered to think more deeply about these inconsistencies, i.e., heuristics and biases, and foster critical thinking (Cohen and Jaffray, 1980; Bell et al., 1988).

Practically (e.g., epistemologically, Creswell, 2014), behavior economy and cognitive psychology propose training techniques are meant to remove the occurrences of mistakes for decision-making process optimization. The underlying idea of this method is to work with individuals' common cognitive errors indicating logical inconsistencies and inconsistent perceptions of reality (Gerling, 2009). Bias removal (Debiasing) programmers are similar to the prescriptive method and the use of reminders, such as warning individuals to consider the base rate of success in the workplace before concluding. Different problems offered by Soll et al. (2014) include training programs in which students share professional experiences about overwhelming errors (to correct underestimation of rare events) and providing new tips and methods for trained employees to critique. Nudging and self-nudging approaches serve for interpreting the multiplicity of these perspectives for the realization of an educational toolkit for enhancing CT and ML in VET students. In this framework, the urgency is to (a) supporting the use of specific procedures to understand whether the information is fake or real; (b) enhancing the awareness of cognitive errors (e.g., cognitive biases) supporting the idea that all people can be irrational, as irrationality is embedded in humans but it can be reduced by the awareness of biases; (c) enhancing the individuals' tools to develop personal skills and procedural activities to address information.

### Self-nudging

The NERDVET community builds up on these previous ontological and epistemological interpretations (Creswell, 2014) and refers to the notion of self-nudging as a general behavioral-cognitive process which can be supported among individuals to help them to reach their targets. The self-nudging is a novel notion whose roots are in the nudge theory. This latter is defined as a strategy to design individuals' choice environments, guiding their behavior to increase wellbeing and work efficacy, productivity, and social engagement (Johnson et al., 2012; Lehner et al., 2016). The impact of nudges is widely recognized, and authors are even more supportive of the idea that individuals can create a simple set of nudges that can be used to apply specific reasoning and behaviors (Thaler and Sunstein, 2008). The self-nudging concept instead is based on the idea of autonomous implementation of nudges, which are non-regulatory and non-monetary strategies that may change the choice architecture to target behavior in a predictable way, toward their ultimate goals, without eliminating any options or significantly changing personal incentives (Thaler and Sunstein, 2008). Nudges are aids or signals that individuals experience all the time: some are designed to initiate or shape new behaviors; others can be used to provide information or guide thinking. In turn, self-nudging is a behavioral science strategy that focuses on individuals' capacity to define a set of strategies to improve their self-control. The idea behind self-nudging is that people can design and structure their environments in ways that make it easier for them to make the right choices and also to reach long-term goals. Also, self-nudging requires consciousness about a connection between one's behavior and the environment's architecture, as well as knowledge of a procedure that can help to modify that connection (Torma et al., 2018).

Considering the congruency of the understanding of CT and ML as two interconnected metacognitive competences (Tommasi et al., 2021a) while assuming the orientation of the self-nudging approach to developing an individual set of behavioral-cognitive strategies (Torma et al., 2018), training bases on eliciting the application of proactive engagement in information processing and also supporting the creation of specific individuals' strategies for critically assessing external information and personal reasoning (Hertwig and Grüne-Yanoff, 2017). By facilitating the use of self-nudge, people have the opportunity, through adequate training, to become architects of their own choices in a fully conscious way. For this reason, it becomes necessary to address people on the causes related to probable behavioral problems and on how to deal with them. According, behavioral researchers are often called to contribute to the change process, but at the same time, they are limited in how they can modify the underlying environment or the entire chosen architecture. Often, they try to push in the context of complex systems in which they can at the most change behavior in a marginal way. Therefore, moving on to reducing “sludge,” like eliminating obstacles that make decision-making difficult, could be more productive, which is indeed the idea behind self-nudging training.

### Self-nudging for CT and ML in VET

By raising awareness about individuals' social responsibility, it becomes possible to improve responsible thinking, critical interpretation, and information comprehension. In an educational context, nudges can thus help increase motivation in students, and encourage them to become more interested and involved in the proposed initiatives; moreover, highly motivated peers contribute to promoting the quality of support and discussion between subjects (Ebert and Freibichler, 2017).

According to this definition, a key factor to enhance CT and ML in VET students is identifying which are the learning purposes associated with self-nudging. Specifically, interventions are meant to develop in subjects the competence to autonomously create nudges for the self, addressed to social responsibility. A significant aspect of this process is the maintenance of motivation, which may aid the growth of critical thinking and media literacy. Another relevant factor is the ability of teachers and trainers of managing to influence the habits of students regarding comprehension and interpretation of information. Those who work in these contexts should pay great attention and provide constant support to any thinking process and proactive behavior.

**Figure 1:**
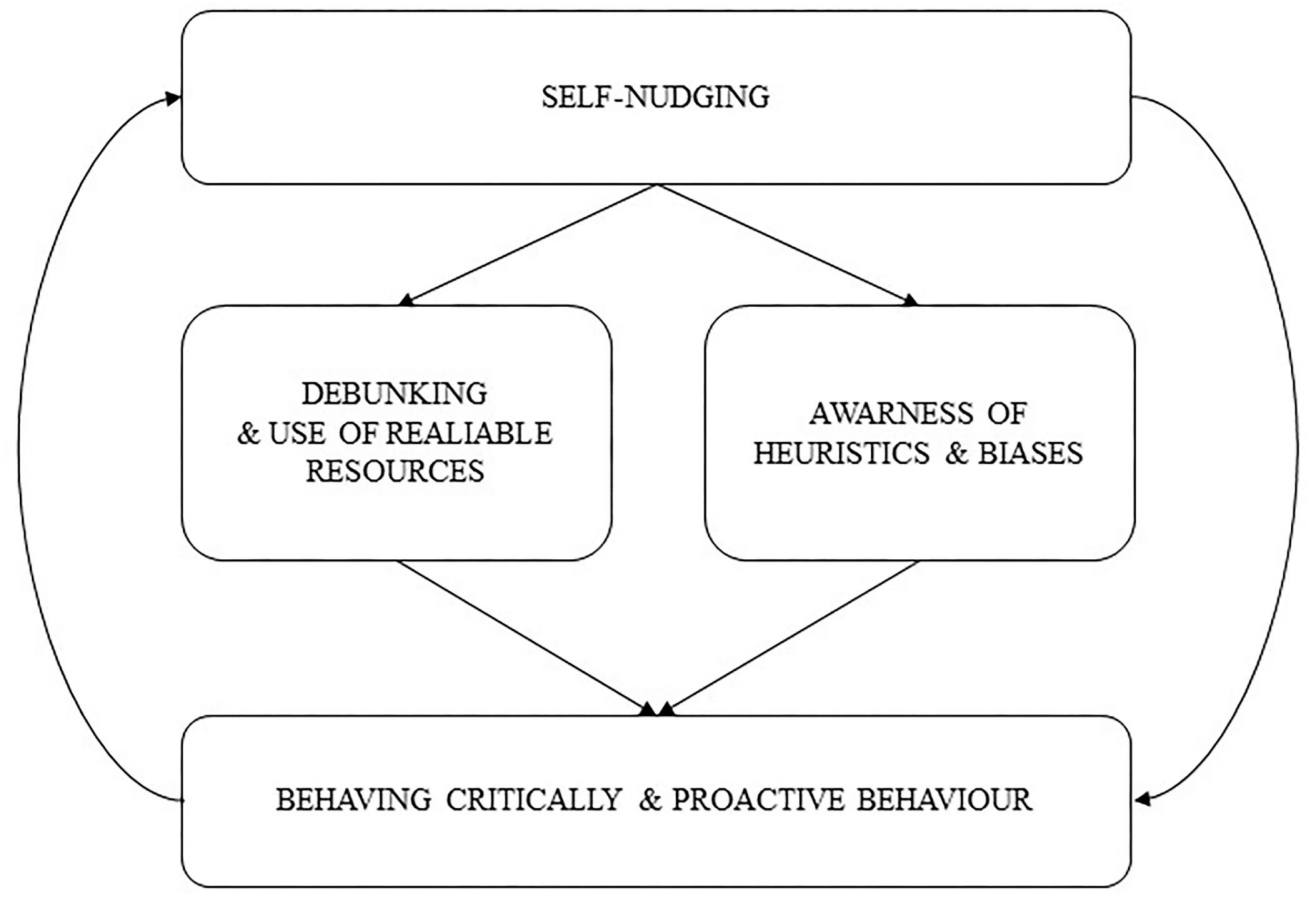
Self-nudging circuit of behaving and thinking critically.

In this framework, we propose that to enhance CT and ML, the teaching staff can follow the self-nudging *circuit of behaving and thinking critically* (Figure 1).

The focus is on eliciting the application of CT and ML by supporting students' proactive engagement in information processing and supporting the creation of specific individuals' strategies for critically assessing information and adopting informed behavior. Teachers and trainers represent one of the key elements for students' creation of their cognitive-behavioral strategies. Thus, the circuit comprehends students' ability to understand the role of personal nudges (cognitive strategies) *via* which activating self-nudging activities influence their behavior. That is, self-nudging as an individual approach to CT and ML leads students to behave critically in a proactive way. In turn, behaving critically and proactively leads to strengthening one's strategies (personal nudges) as well as creating new ones. As noted, individuals can themselves create a simple set of nudges that can be used to apply specific reasoning and behaviors (Thaler and Sunstein, 2008).

#### Self-nudging techniques

In the digital era, the problem of fake news affects both the quantitative and qualitative aspects of the information environments. The challenge of recognizing fake news worldwide can be addressed by developing learners' ability to evaluate and chose better sources of information. In these terms, self-nudging can focus on supporting the use of debunking strategies and the use of reliable sources. This means teaching students how to understand whether the information is fake or real by enhancing their awareness of the relevance of the sources. Thus, teachers can help students to realize specific strategies to control the source of data in reading and choosing information. An efficient way for developing these self-nudging strategies can be supporting the use of debunking by providing resources for students to debunk, such as a guide on how to flag suspicious stories on social media networks and a list of websites that have carried false or satirical articles. Alongside, this approach should also include training on the use of reliable sources, which means teaching students how and to what extent sources of information matter and how to find good sources.

To behave and think critically, individuals also need to avoid their prejudices and irrational beliefs, which may be due to the use of specific cognitive shortcuts, i.e., heuristics and biases. In these terms, the main aspect of reducing the incidence of cognitive biases and prejudices (i.e., irrational beliefs) is to develop awareness about them. Teaching people what cognitive biases are, how to recognize them, and what their effects can be, help reduce their incidence but also, in the view of self-nudging, help support the creation of specific strategies to avoid their use in fast reasoning. As already mentioned, CT is also related to addressing irrational beliefs (i.e., stimulating emotional strategies), which are usually connected to prejudice and emotional judgments (Kahneman, 2003). Applying self-nudging to counter bad heuristics processes could result in both stopping them and creating good ones: in this context, some examples of nudges could be forcing oneself to consider more options, using checklists, activating reminders, and learning to practice rewording in the presence of ambiguous information (Orosz et al., 2016).

Finally, another relevant aspect related to CT is the ability to break down information or problems to solve a small problem or analyze a simpler piece of information at a time. Using self-nudging in this framework would mean helping people to foster their abilities to practice continuous but simpler problem-solving and to constantly check their activities against a prior detailed plan they had formulated.

### Direct users and beneficiaries

Following our model, teachers and trainers represent one of the key targets as direct users of the NERDVET approach. The contemporary setup of VET contexts underlines (a) the need for VET teachers and trainers to equip students with critical thinking skills and media literacy as well as (b) the lack of formal training paths on the identified topics, thus supporting teachers and trainers in empowering students to become the future generation of EU citizens. The role teachers and trainers can play has crucial importance as long as they are (1) better equipped and trained, (2) well-aware of the direct benefits for and impact on students, (3) capitalizing on the expertise developed at the European and national levels by other VET peers, having direct access to successful practices and teaching and training methods.

Furthermore, the upskilling proposal is expected to have a direct impact on iVET students as beneficiaries of the NERDVET approach. Motivation and active engagement are two key factors for a successful teaching and learning process, especially in the iVET sector where major challenges derive from the disadvantaged socioeconomic background owned by students. In fact, these pre-conditions affect not only students' performance but also their willingness to contribute to the overall learning process proposed by teachers and trainers. Following a motivational-oriented approach and applying active teaching methods, the target is to put students at the very center of the learning process with a double scope: (1) to equip iVET students with both technical and soft skills that are considered crucial to entering the labor market, (2) to allow teachers or trainers to be perceived as proactive actors–mentors and not as mere instructors–who are capable to turn teaching into a mutual learning process, where dialogue and support pave the way for students' personal and professional development.

### Evaluating the NERDVET approach

The NERDVET project is administrated by teachers and researchers to propose forward normative instruments and projects characterized by high effectiveness to promote students learning. Hence, the evaluation of the NERDVET approach should focus on the outcomes of students and teachers involved in the training activities. A valuable pathway for the evaluation of the effectiveness of the NERDVET approach to enhance CT and ML should be based on the use of mix-methods involving qualitative interviews and quantitative instruments for students' performance assessment (Sartori and Ceschi, 2013; Creswell, 2014). First, a qualitative evaluation approach could be based on Vergani's (2004) core-methodological perspective. Vergani proposed a system of qualitative evaluation based on viewing the prospects of participants in training activities and creating the evaluation system itself. He suggested considering the point of view of the evaluated rather than applying a standardized system and making inferences on the effectiveness of the training programs (2004). By the explicit comparison between all the data collected, i.e., documents, and interviews, the evaluation of a training project can emerge and core aspects of the students' competence development. Hence, researchers could collect qualitative data after the training in class and in the workplace using interviews coupled with document analysis. Students and teachers can be interviewed with a semi-structured questionnaire about their experience in the study. Moreover, qualitative methods aim to understand the features of the project as a result of the experiential contents, and the comparison with the documents made by teachers and authorities involved.

Second, quantitative methods can be used to assess students' and teachers' competence development (Sartori et al., 2015). To pursue this aim, researchers can develop a new self-report questionnaire for teachers and students. We suggest the use of a self-evaluation tool for allowing teachers and trainers to verify their level of CT-ML, whether there is room for improvement, and whether the task was effective for the learning process. Results would be easily interpreted by looking at the range of the answers by assessing students before and after training to have a quantitative indication of their improvement in CT and ML.

## Conclusion

We proposed this contribution to offer initial suggestions on the possibility of applying a novel educational approach. This limits this work to a mere presentation of the state of the art of literature on CT and ML in the context of VET, and the self-nudging approach realized by the NERDVET project. In this framework, our ultimate aim is to offer a link between the social need for the promotion of CT and ML and the progress of research in cognitive-behavioral psychology. Considering the actual limitations in promoting CT and ML in the VET world, this work represents the first step, by setting a theoretical approach that can be further developed and implemented in educational programs. Indeed, the present contribution contains both a terminological orientation and a general depiction of the possibilities of using self-nudging in VET for promoting CT and ML. Future applied research activities may use this work as a starting point to devise training interventions. Similarly, this study can lead to realizing policy recommendations and novel educational frameworks in the field of VET.

## Data availability statement

The original contributions presented in the study are included in the article/supplementary material, further inquiries can be directed to the corresponding author.

## Author contributions

FT and RS are responsible for the title, the abstract, the general idea of the paper, and wrote the manuscript. FT, RS, AC, and MF developed the concept behind the manuscript. SG and SB provided the literature sources and contributed to the design of the model. AC, MF, SG, and SB edited the final version of the manuscript. MF is, in particular, responsible for the introduction and the conclusion. All authors contributed to the elaboration of the concept to a publishable topic, have made a substantial, direct and intellectual contribution to the work, and approved it for publication.

## Funding

Authors' work on this paper was supported by of the European Union funding for the project NERDVET, ERASMUS + KA3 - Support for Policy Reform, Social inclusion through education, training, and youth.

## Conflict of interest

The authors declare that the research was conducted in the absence of any commercial or financial relationships that could be construed as a potential conflict of interest.

## Publisher's note

All claims expressed in this article are solely those of the authors and do not necessarily represent those of their affiliated organizations, or those of the publisher, the editors and the reviewers. Any product that may be evaluated in this article, or claim that may be made by its manufacturer, is not guaranteed or endorsed by the publisher.

## Footnotes

^1^NERDVET - Think smart! Enhancing critical thinking skills and media literacy in VET - Project no. 621537-EPP-1-2020-1-IT-EPPKA3-IPI-SOC-IN | Erasmus+ KA3 - Support for policy reform - Call EACEA/34/2019. Website: https://www.nerdvet.eu/.
